# Extended GLRT Detection of Moving Targets for Multichannel SAR Based on Generalized Steering Vector

**DOI:** 10.3390/s21041478

**Published:** 2021-02-20

**Authors:** Chong Song, Bingnan Wang, Maosheng Xiang, Wei Li

**Affiliations:** 1 National Key Laboratory of Microwave Imaging Technology, Aerospace Information Research Institute, Chinese Academy of Sciences, Beijing 100094, China; songchong18@mails.ucas.edu.cn (C.S.); xms@mail.ie.ac.cn (M.X.); liwei199@mails.ucas.ac.cn (W.L.); 2 School of Electronics, Electrical and Communication Engineering, University of Chinese Academy of Sciences, Beijing 100094, China

**Keywords:** generalized likelihood ratio test (GLRT), GLRT based on the generalized steering vector (GSV-GLRT), ground moving target indication (GMTI), multichannel synthetic aperture radar (MSAR)

## Abstract

A generalized likelihood ratio test (GLRT) with the constant false alarm rate (CFAR) property was recently developed for adaptive detection of moving targets in focusing synthetic aperture radar (SAR) images. However, in the multichannel SAR-ground moving-target indication (SAR-GMTI) system, image defocus is inevitable, which will remarkably degrade the performance of the GLRT detector, especially for the lower radar cross-section (RCS) and slower radial velocity moving targets. To address this issue, based on the generalized steering vector (GSV), an extended GLRT detector is proposed and its performance is evaluated by the optimum likelihood ratio test (LRT) in the Neyman-Pearson (NP) criterion. The joint data vector formulated by the current cell and its adjacent cells is used to obtain the GSV, and then the extended GLRT is derived, which coherently integrates signal and accomplishes moving-target detection and parameter estimation. Theoretical analysis and simulated SAR data demonstrate the effectiveness and robustness of the proposed detector in the defocusing SAR images.

## 1. Introduction

As an advanced modern sensor that allows large area coverage in all-weather conditions during day and night, synthetic aperture radar (SAR) is widely applied in both civil and military fields [1,2]. Synthetic aperture radar-ground moving-target indication (SAR-GMTI), which combines SAR imaging and GMTI technology, has played an important role in the modern radar system. Due to the increase of the spatial degree of freedom, multichannel SAR (MSAR) can address the limit of velocity detection and the strong clutter interference in single-channel SAR [3,4]. It can significantly improve the performance of moving-target detection, especially the ability to detect slow-moving targets.

SAR-GMTI algorithms can roughly be categorized into two classes. The first works on the raw data, and the second works on the processed SAR image. The SAR-GMTI algorithm we propose works on the processed SAR image. By jointly exploiting the space–time information, conventional space–time adaptive processing (STAP) methods are applied on range-compressed raw data and suppress clutter in the time domain [5] or frequency domain [6,7]. Usually, the coherent processing interval (CPI) should be chosen to ensure that a moving target stays in one range-Doppler cell but this may suffer signal-to-noise ratio (SNR) loss [8]. There are also some other problems such as the computational burden and training data size [9]. In [8], the proposed imaging STAP (ISTAP) overcomes this problem and can detect slow-moving targets. In [10], the proposed post-Doppler parametric adaptive matched filter (PD-PAMF) models the clutter as an auto-regressive (AR) process for filtering, which has lower requirements of training data size. In the complex image domain, the traditional displaced phase center antenna (DPCA) exploits both the phase and the amplitude differences of SAR image sequences to detect moving targets [11,12]. The detection performance of DPCA is greatly affected by the SNR. When the SNR is significantly reduced, its detection performance worsens [13,14]. Traditional along-track interferometry (ATI) only exploits the phase difference of SAR image sequences and it does not carry out clutter cancellation [11]. As for a lower signal-to-clutter ratio (SCR), the detection performance degrades rapidly [14,15,16]. DPCA and ATI were originally designed for two-channel systems and are suboptimum when more than two channels are available. In [17], a generalized DPCA (EDPCA) is proposed, which can effectively detect moving targets and accurately estimate the parameters of moving targets. DPCA, ATI and EDPCA exploit the information contained in the current range-azimuth pixel only for moving-target detection. As a result, their performance is easily affected by the quality of complex SAR image sequences. Generally speaking, compared with the methods in the raw data domain, better performance can be obtained in the image domain due to the fact that the moving targets can be partially coherently integrated via the azimuth focusing [18].

Unfortunately, in SAR-GMTI systems, because of the relative motion between targets (i.e., moving targets and stationary targets) and platform during the synthetic aperture time, it is inevitable that defocusing will result in the imaging process [18,19]. Especially for moving targets, the Doppler mismatch caused by their 2-dimensional (2-D) motions and the uncompensated phase modulation will aggravate the defocus [20,21,22,23]. Moreover, in a complex environment, various factors such as aircraft undesirable movement, velocity misalignment and clutter internal motion will manifest as image defocus. The defocusing effect usually degrades the detector’s performance. In addition, SAR systems are often oversampled and use matched filters in the imaging process, so the adjacent pixels are statistically dependent [24].

The generalized likelihood ratio test (GLRT) detector has the constant false alarm rate (CFAR) property so that the false alarm probability is independent of the clutter covariance matrix [25,26]. The distribution (clutter and noise) seriously affects the detection of the moving targets. In general, the detectors need to suppress the distribution and then achieve the detection and parameter estimation [11,27]. However, for the GLRT detector, since the distribution is taken into account in the statistical signal model and assumed a fixed clutter covariance matrix, target detection and parameter estimation can be accomplished while the distribution is suppressed. In some cases, the operation without the preliminary distribution cancellation can improve the efficiency of calculation and avoid the SNR loss caused by clutter suppression, which is important for detecting smaller and slower targets. In [27,28], Budillon had demonstrated the effectiveness of the GLRT detector via multibaseline along-track interferometric SAR systems.

However, the defocusing effect of the images usually produces a loss in the signal intensity, which severely degrades the detection performance of the conventional GLRT. Especially, for the targets with slower radial velocity and lower radar cross-section (RCS), this effect may invalidate the conventional GLRT detector. To overcome the performance loss in the defocusing SAR images, we propose an extended GLRT for the multichannel SAR. Since the adjacent cells are correlative, we use the current cell and the adjacent cells to form the joint data vector, named the generalized steering vector (GSV), and the the joint covariance matrix. The target is adopted by a deterministic model and the clutter is assumed as a Gaussian model. Based on the GSV, the extended GLRT is derived. The detector accumulates the dispersed energy of the targets while the distribute is suppressed, and then achieves target detection. It is demonstrated here that the proposed detector offers dramatic improvement in detection performance over the conventional GLRT. Furthermore, it reaches an accurate estimation of the target radial velocity.

The outline of this paper is as follows. In Section 2, the single-pixel signal statistical model and traditional GLRT are introduced. In Section 3, the statistical model of a joint pixel signal based on the generalized steering vector is introduced, and the extended GLRT based on the generalized steering vector and its optimal performance are derived in detail. In Section 4, the validation of the proposed detector has been carried out by theoretical analysis and simulated SAR data. Section 5 presents some discussions and Section 6 contains our conclusions.

Vectors (matrices) are denoted by boldface lower(upper) case letters. ${\left[\cdot \right]}^{T}$ denotes matrix transpose. ${\left[\cdot \right]}^{H}$ denotes conjugate transpose of matrix. E[·] is the expectation operator. |·| represents the determinant of the matrix argument, and |·| with a complex number represents the modulus. Other notation usage is introduced in-place.

## 2. Multichannel Signal Statistical Model and GLRT Overview

**Figure 1:**
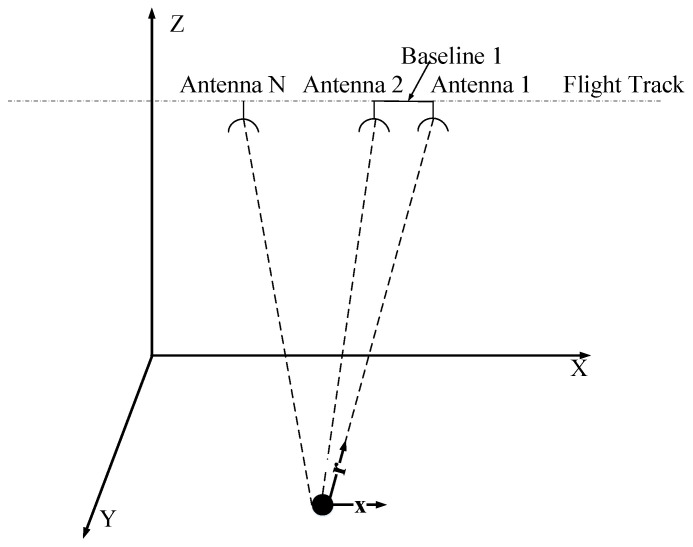
Multichannel synthetic aperture radar (MSAR) system geometry.

In this section, we review the single-pixel signal statistical model for MSAR and the traditional GLRT detector in the complex image domain [18,27,29,30]. Assume that the side-looking SAR-GMTI system has N channels in azimuth. The first channel serves as both a transmitting and receiving channel, which is known as a reference channel, and the other channels only receive echoes, which are known as an auxiliary channel. The effective baseline length of the nth channel is $b_{n}$, with n = 1, *…*, N and $b_{1}=0$. Assume b≪Ha, which is the platform distance from the ground. A moving target on the ground with a constant velocity $V_{t}=V_{x}x+V_{r}r$. **x** is the unit vector of azimuth and **r** is the unit vector of range. $V_{x}$ and $V_{r}$ denote the azimuth and range velocity components, respectively. The distance between the two channels is the physical baseline, as shown in Figure 1.

Let $z\left(i\right)={\left[z_{1}\left(i\right),z_{2}\left(i\right),\ldots ,z_{N}\left(i\right)\right]}^{T}$ represent complex N-dimensional vectors. The binary hypothesis can be written as follows [28]:

(1) $$\left\{\begin{array}{c} H_{0}:z\left(i\right)=c\left(i\right)+w\left(i\right)\\ H_{1}:z\left(i\right)=s\left(i\right)+c\left(i\right)+w\left(i\right) \end{array}\right.$$

where $H_{0}$ is in absence of a moving target, and $H_{1}$ is in presence of a moving target. $c\left(i\right)={\left[c_{1}\left(i\right),c_{2}\left(i\right),\ldots ,c_{N}\left(i\right)\right]}^{T}$ denotes the steering vector of the clutter. $w\left(i\right)={\left[w_{1}\left(i\right),w_{2}\left(i\right),\ldots ,w_{N}\left(i\right)\right]}^{T}$ denotes the steering vector of the noise.They can be assumed as circularly symmetric complex Gaussian vectors, with mutually uncorrelated real and imaginary parts, with zero mean and same variance ($\frac{\sigma_{c}^{2}}{2}$ and $\frac{\sigma_{n}^{2}}{2}$). Under $H_{0}$ , the clutter-plus-noise covariance matrix is given by $R\left(i\right)=E[z\left(i\right)z^{H}\left(i\right)]$. s(i) denotes the vector of the moving target based on a deterministic target model.

(2) $$s\left(i\right)=\alpha_{i}p\left(i\right)\;n=1,\ldots ,N$$

where $\alpha_{i}$ is the complex target reflectivity image of pixel *i* and $p\left(i\right)={\left[e^{j\phi_{i1}},e^{j\phi_{i2}},\ldots ,e^{j\phi_{iN}}\right]}^{T}$ represents the steering vector of the target signal. $\phi_{in}$ is the phase shift between the first channel and nth channel. As the maximum size of the observed scene is small compared with the antenna distance to the target, the phase shift can be expressed as follows [19]:

(3) $$\phi_{in}=\frac{4\pi b_{n}V_{r}}{\lambda V}\;n=1,\ldots ,N$$

where λ is the wavelength of the SAR system and V is the platform velocity. The probability density function (PDF) can be modeled as a multi-dimensional complex Gaussian distribution with a covariance matrix of R(i). Thus, under the condition of $H_{0}$, the PDF can be written as [30]:

(4) $$p_{z}\left(z|H_{0}\right)=\frac{1}{\pi^{N}\left|R\right|}\cdot e^{-z^{H}R^{-1}z}$$

Under the condition of $H_{1}$, the PDF can be written as [30]:

(5) $$p_{z}\left(z|\alpha ,V_{r},H_{1}\right)=\frac{1}{\pi^{N}\left|R\right|}\cdot e^{-{\left(z-s\right)}^{H}R^{-1}\left(z-s\right)}$$

For simplicity, the symbol *i* has been omitted. |R| is the determinant of the matrix argument. Based the aforementioned PDFs, the test variable of the GLRT detector can be expressed as follows [27]:

(6) $$\Lambda_{GLRT}\left(z\right)=\frac{{}_{\alpha ,V_{r}}^{max}p_{z}\left(z|\alpha ,V_{r},H_{1}\right)}{p_{z}\left(z|H_{0}\right)}\overset{H_{0}}{\underset{H_{1}}{{}_{>}^{<}}}\gamma$$

where γ is the threshold for detection and it can be determined by CFAR.

The traditional GLRT bases on a single-pixel model to detect the moving targets. When the SAR image is defocusing, the energy of the target will be split into multiple pixel cells. Therefore, single-pixel detection inevitability causes serious performance loss, which may lead to the low RCS moving target not being identified at all. The application of a single-pixel pair is equivalent to using only spatial degrees of freedom. Obviously, the anti-jamming ability is poor. A feasible method is to combine multiple pixels to effectively accumulate the energy and then achieve detection. The proposed method in this paper is built on this idea, which will be introduced in detail in Section 3.

## 3. Extended GLRT Model Based on Generalized Steering Vector

When the SAR images were fully focused, this indicated the GLRT achieved good performance. However, in SAR-GMTI systems, it is not realistic. A defocusing effect produces a loss in the signal intensity, which may remarkably degrade the detector performance. In this paper, we propose a method, based on the generalized steering vector model, to effectively overcome the problem of moving-target detection resulting in defocused SAR images.

This section mainly introduces the joint pixel signal statistical model and the extended GLRT based on the generalized steering vector (GSV-GLRT). Futhermore, GSV-GLRT utilizes the current pixel and its adjacent pixels to accumulate the target signal coherently and increase the temporal degrees of freedom, which improves the detectability of moving targets . According to the Neyman–Pearson (NP) criterion [31], the optimal detector is the LRT, but it cannot be implemented in practice [32]. GLRT facilitates replacement of the unknown parameters with their maximum likelihood estimates under each hypothesis based on the entirety of data, which is suboptimal [32]. However, we also derive the optimal LRT based on the generalized steering vector (GSV-LRT) as a best performance reference of GSV-GLRT.

### 3.1. Modified Signal Statistical Model

**Figure 2:**
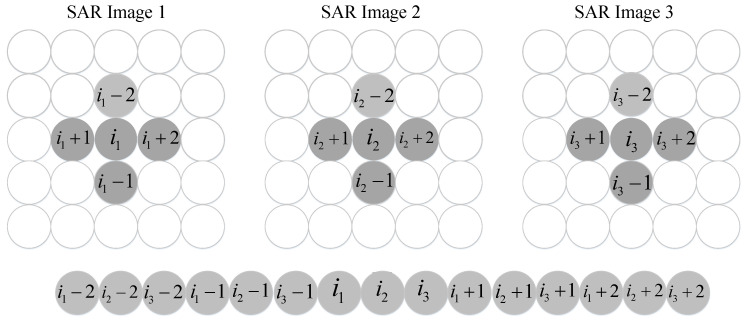
Formulation of joint data vector.

By considering the defocusing characteristics of moving targets and the correlation of adjacent pixels in the SAR image, we use the current pixel and its adjacent pixels to construct the joint data vector for GLRT detection. Without losing generality, we chose radar with three channels and four adjacent pixels like “+” windows for modelling. When images are severely defocused, we should expand the windows; however, this will increase the calculation burden. However, how to choose the appropriate windows is outside the scope of this article. The formulation of the joint data vector is shown in Figure 2.

The binary hypothesis can be written as follows:

(7) $$\left\{\begin{array}{c} H_{0}:z_{J}\left(i\right)=c_{J}\left(i\right)+w_{J}\left(i\right)\\ H_{1}:z_{J}\left(i\right)=s_{J}\left(i\right)+c_{J}\left(i\right)+w_{J}\left(i\right) \end{array}\right.$$

where $H_{0}$ is in absence of a moving target, and $H_{1}$ is in presence of a moving target.

(8) $$z_{J}\left(i\right)={\left[z^{T}\left(i-2\right),z^{T}\left(i-1\right),z^{T}\left(i\right),z^{T}\left(i+1\right),z^{T}\left(i+2\right)\right]}^{T}$$

where $z_{J}\left(i\right)$ is the joint data vector.

(9) $$\begin{array}{ccc} s_{J}\left(i\right) & = & {\left[\beta_{i-2}s^{T}\left(i-2\right),\beta_{i-1}s^{T}\left(i-1\right),\beta_{i}s^{T}\left(i\right),\beta_{i+1}s^{T}\left(i+1\right),\beta_{i+2}s^{T}\left(i+2\right)\right]}^{T} \end{array}$$

where

(10) $$\beta_{m}=\frac{E[z\left(i\right)z^{*}\left(m\right)]}{\sqrt{{E[|z\left(i\right)|}^{2}{]E[|z\left(m\right)|}^{2}]}}\;m=i-2,\ldots ,i+2$$

$s_{J}\left(i\right)$ is the moving-target vector. ${\left[\right]}^{*}$ denotes conjugate.

(11) $$\beta ={\left[\beta_{i-2},\beta_{i-1},\beta_{i},\beta_{i+1},\beta_{i+2}\right]}^{T}⊗1_{N}$$

where β is the correlation factor vector, which is determined by the quality of SAR focus. We obtain β from the correlation of adjacent pixels. $1_{N}$ is the all ones N-dimensional column vector. ⊗ denotes the Kronecker product.

(12) $$p_{J}\left(i\right)=\beta ⊙{\left[p^{T}\left(i\right),p^{T}\left(i\right),p^{T}\left(i\right),p^{T}\left(i\right),p^{T}\left(i\right)\right]}^{T}$$

where p(i) is the target steering vector, as defined in Equation (2). $p_{J}$ is called the generalized steering vector [33]. ⊙ denotes the Hadamard product. $R_{cJ}\left(i\right)=E\left[c_{J}\left(i\right)c_{J}^{H}\left(i\right)\right]$ is the joint covariance matrix of the clutter, whose element value is determined by the clutter power and the correlation between the current pixel and its adjacent pixels. Under $H_{0}$, the corresponding clutter-plus-noise covariance matrix is given by $R_{J}\left(i\right)=E\left[z_{J}\left(i\right)z_{J}^{H}\left(i\right)\right]$. In this paper, we assume $R_{J}\left(i\right)$ is known, and, of course, it can be estimated from the secondary data [17,34,35,36]. According to the aforementioned signal model, under the condition of $H_{0}$, the PDF can be written as follows:

(13) $$p_{z_{J}}\left(z_{J}|H_{0}\right)=\frac{1}{\pi^{N}\left|R_{J}\right|}\cdot e^{-z_{J}^{H}R_{J}^{-1}z_{J}}$$

Under the condition of $H_{1}$, the PDF can be written as follows:

(14) $$p_{z_{J}}\left(z_{J}|\alpha ,V_{r},H_{1}\right)=\frac{1}{\pi^{N}\left|R_{J}\right|}\cdot e^{-{\left(z_{J}-s_{J}\right)}^{H}R_{J}^{-1}\left(z_{J}-s_{J}\right)}$$

### 3.2. Extended GLRT Based on Generalized Steering Vector Derivation

The moving-target parameters are modeled as deterministic unknown parameters and are replaced with their ML-estimate in the GLRT. With this strategy, the GSV-GLRT can be expressed as follows:

(15) $$\Lambda_{GSV-GLRT}\left(z_{J}\right)=\frac{{}_{\alpha ,V_{r}}^{max}p_{z_{J}}\left(z_{J}|\alpha ,V_{r},H_{1}\right)}{p_{z_{J}}\left(z_{J}|H_{0}\right)}\overset{H_{0}}{\underset{H_{1}}{{}_{>}^{<}}}\gamma$$

We can now take the logarithm, and simplify to:

(16) $$\begin{array}{ccc} \ln(\Lambda_{GSV-GLRT}\left(z_{J}\right)) & = & 2R\left(\alpha^{*}p_{J}^{H}R_{J}^{-1}z_{J}\right)-{\left|\alpha \right|}^{2}p_{J}^{H}R_{J}^{-1}p_{J} \end{array}$$

R() is getting the real part of complex data. Maximizing Equation (16) with respect to the unknown complex amplitude α yield:

(17) $$\alpha =\frac{p_{J}^{H}R_{J}^{-1}z_{J}}{p_{J}^{H}R_{J}^{-1}p_{J}}$$

Then the GSV-GLRT is obtained:

(18) $$\Lambda_{GSV-GLRT}^{{}^{\prime }}\left(z_{J}\right)={}_{V_{r}}^{max}\frac{|p_{J}^{H}R_{J}^{-1}z_{J}{|}^{2}}{p_{J}^{H}R_{J}^{-1}p_{J}}\overset{H_{0}}{\underset{H_{1}}{{}_{>}^{<}}}\gamma^{{}^{\prime }}$$

Usually, the test statistic $\Lambda_{GSV-GLRT}^{{}^{\prime }}\left(z_{J}\right)$ is obtained by searching a set of possible values of the $V_{r}$. Therefore, the Equation (18) is not a closed form. Fortunately, we can use the Monte Carlo simulation to obtain the false alarm probability $\left(P_{FA}\right)$, the detection probability $\left(P_{D}\right)$ and the receiver operating characteristic (ROC) in order to evaluate the detection performance of the GSV-GLRT. Moreover, the covariance matrix $R_{J}$ is usually accounted for by using adaptive techniques, which is independent of the actual clutter covariance matrix [26,34]. This operation displays the CFAR property.

Compared with traditional GLRT, GSV-GLRT not only uses GSV to coherently accumulate target signal, but also utilizes clutter-plus-noise joint covariance matrix to suppress clutter, which greatly improves the detection performance of the system. At the same time, making good use of multiple pixels is equivalent to increasing the temporal degrees of freedom, so the adaptive detection ability is also robust in non-ideal conditions. The multi-pixel joint processing can also effectively suppress sidelobe clutter, which has an influence on moving-target detection [37].

### 3.3. Optimal Detection Performance Analysis

Assuming that the parameters of the moving target are known, the GSV-LRT is given as follows:

(19) $$\Lambda_{GSV-LRT}\left(z_{J}\right)=\frac{p_{z_{J}}\left(z_{J}|\alpha ,V_{r},H_{1}\right)}{p_{z_{J}}\left(z_{J}|H_{0}\right)}\overset{H_{0}}{\underset{H_{1}}{{}_{>}^{<}}}\gamma$$

Taking the logarithm and performing some simplification, it leads to the following:

(20) $$\Lambda_{GSV-LRT}^{{}^{\prime }}\left(z_{J}\right)=R\left(s_{J}^{H}R_{J}^{-1}z_{J}\right)$$

where

(21) $$\gamma^{{}^{\prime }}=\frac{1}{2}\left(\gamma^{{}^{\prime }}+s_{J}^{H}R_{J}^{-1}s_{J}\right)$$

The detection performance ($P_{FA}$ and $P_{D}$) is determined by the PDF of $\Lambda_{GSV-LRT}^{{}^{\prime }}\left(z_{J}\right)$ under H0 and H1.

#### 3.3.1. $P_{FA}$ under H0

As $z_{J}∼CN\left(0,R_{J}\right)$, we have

(22) $$\sigma^{2}=var\left(\Lambda_{GSV-LRT}^{{}^{\prime }}\left(z_{J}\right)\right)=\frac{s_{J}^{H}R_{J}^{-1}s_{J}}{2}$$

(23) $$\Lambda_{GSV-LRT}^{{}^{\prime }}\left(z_{J}\right)=R\left(s_{J}^{H}R_{J}^{-1}z_{J}\right)∼CN\left(0,\sigma^{2}\right)$$

The corresponding value of $P_{FA}$ is given by the following:

(24) $$P_{FA}=Pr\left\{\Lambda_{GSV-LRT}^{{}^{\prime }}\left(z_{J}\right)\geq \gamma^{{}^{\prime }};H_{0}\right\}=Q\left(\frac{\gamma^{{}^{\prime }}}{\sigma }\right)$$

The threshold is:

(25) $$\gamma^{{}^{\prime }}=\sigma Q^{-1}\left(P_{FA}\right)$$

where $Q\left(x\right)=\int_{x}^{+\infty }\frac{1}{\sqrt{2\pi }}e^{-\frac{1}{2}t^{2}}dt$, and $Q^{-1}\left(x\right)$ is the inverse integral function.

#### 3.3.2. $P_{D}$ under H1

As $z_{J}∼CN\left(s_{J}^{H}R_{J}^{-1}s_{J},R_{J}\right)$, we have

(26) $$\Lambda_{GSV-LRT}^{{}^{\prime }}\left(z_{J}\right)=R\left(s_{J}^{H}R_{J}^{-1}z_{J}\right)∼CN\left(2\sigma^{2},\sigma^{2}\right)$$

The corresponding value of $P_{D}$ is given by the following:

(27) $$P_{D}=Pr\left\{\Lambda_{GSV-LRT}^{{}^{\prime }}\left(z_{J}\right)\geq \gamma^{{}^{\prime }};H_{1}\right\}=Q\left(\frac{\gamma^{{}^{\prime }}-2\sigma^{2}}{\sigma }\right)$$

The ROC is:

(28) $$P_{D}=Q\left(Q^{-1}\left(P_{FA}-2\sigma \right)\right)$$

Since GSV-LRT is optimal in the Neyman–Pearson criterion, Equation (28) can be used as a reference for assessing the performance of GSV-GLRT.

## 4. Numerical Results

To assess the detection and estimation performance of the proposed GSV-GLRT, we presented the theoretical performance and the experimental results of simulated SAR data. In this section, performance factors are analyzed, including the quality of SAR image focus and SCR, and then the minimum detectable velocity (MDV) and the Cramer Rao Lower Bounds (CRLBs) for the radial velocity estimation are evaluated. Finally, the experiment of simulated SAR Data is carried out. In the different experiments, the performance of the GSV-GLRT is compared with the traditional GLRT. GSV-LRT and LRT are used as the optimal performance reference of GSV-GLRT and GLRT, respectively.

### 4.1. Theoretical Performance

The theoretical performance of the GSV-GLRT was studied via computer simulation. In other words, using the Monte Carlo simulation method to evaluate the estimation of the detection probability ($P_{D}$) and velocity under different conditions. In this paper, we use a linear uniform array radar of three channels to perform the experiments. The antenna separation was *b* = 0.3 m, and the corresponding effective baseline was $b_{n}$ ($b_{n}=\frac{(n-1)b}{2},n=1,2,3$). The wavelength was 0.03 m, the platform velocity was 100 m/s, and the pulse repetition frequency (PRF) was 1000 Hz.

#### 4.1.1. Receiver Operating Characteristic (ROC) Curves

**Figure 3:**
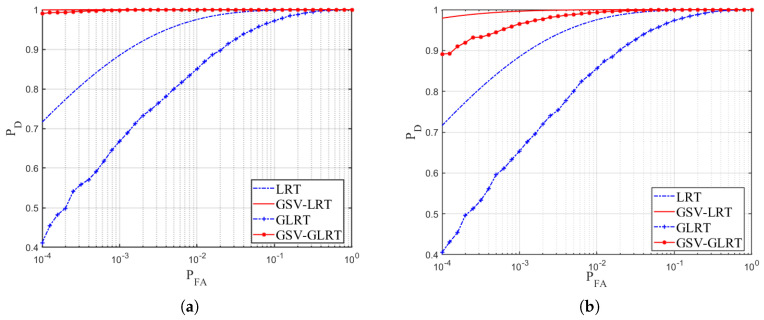

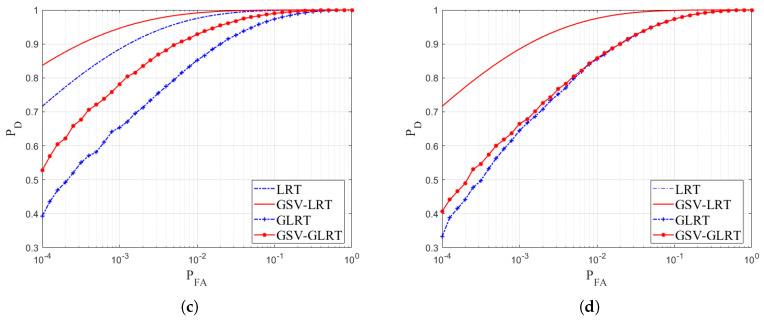
Receiver Operating Characteristic (ROC) curves, $V_{r}$ = 3 m/s, signal-to-clutter ratio (SCR) = –5 dB, clutter-to-noise ratio (CNR) = 10 dB, $P_{FA}=10^{-4}$, three channels, b1 = 0 m, b2 = 0.15 m, b3 = 0.3 m, (**a**) $\beta ={\left[0.8,0.8,1,0.4,0.4\right]}^{T}⊗1_{3}$, high defocus and high neighborhood correlation (**b**) $\beta ={\left[0.6,0.6,1,0.2,0.2\right]}^{T}⊗1_{3}$, moderate defocus and moderate neighborhood correlation (**c**) $\beta ={\left[0.3,0.3,1,0.1,0.1\right]}^{T}⊗1_{3}$, slight defocus and slight neighborhood correlation (**d**) $\beta ={\left[0,0,1,0,0\right]}^{T}⊗1_{3}$, full focus and neighborhood irrelevant.

Figure 3 shows the ROC curves for different focus qualities. The ROC curves were obtained through a Monte Carlo experiment of $10^{5}$ repetitions. The simulation parameter SCR was −5 dB. We assume that the SCR of the current pixel was constant. The clutter-to-noise ratio (CNR) was 10 dB. The GSV-LRT (red solid line) was used as the best performance reference curve for the GSV-GLRT (red dot solid line). The LRT (blue dashed line) was used as the best performance reference curve for the traditonal GLRT (blue plus dashed line). The curves of the GSV-GLRT and GLRT were obtained by searching over the range of the target velocity, and the curves of the GSV-LRT and LRT were obtained by the known target velocity. As we can see, compared with the detection performance of known target parameters, both GSV-GLRT and GLRT have a certain performance loss, which was caused by their parameter estimation step in Equation (18) and Equation (6). For Figure 3a–d, the correlation factor was from the high defocus to the full focus. When the images are focusing, the GSV-GLRT shows the same performance as the GLRT, as shown in Figure 3d. When images defocus, the GSV-GLRT offers a dramatic improvement in detection performance over the conventional GLRT. Especially, in Figure 3a, it can be seen that the detection probability of the GSV-GLRT increased by approximately 0.6 with the $P_{FA}=10^{-4}$. Therefore, the proposed GSV-GLRT outperforms the GLRT.

#### 4.1.2. Detection Probability Versus SCR

**Figure 4:**
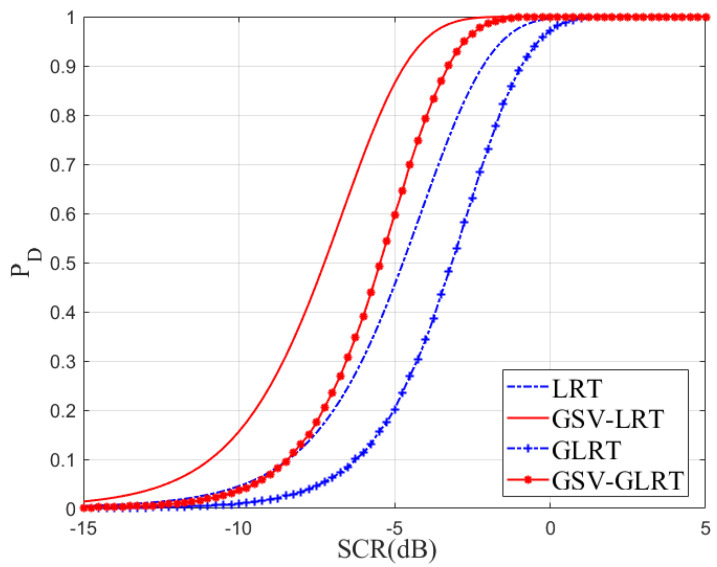
Detection probability versus SCR, $V_{r}$= 2 m/s, CNR =10 dB, $P_{FA}=10^{-4}$ ,three channels, b1 = 0 m, b2 = 0.15 m, b3 = 0.3 m, $\beta ={\left[0.6,0.6,1,0.2,0.2\right]}^{T}⊗1_{3}$.

Then, in Figure 4, the effect of SINR on $P_{D}$ is shown. The $P_{FA}$ was chosen as under $10^{-4}$. The curves were drafted by using $10^{5}$ repetitions. The parameter estimation step also caused some performance loss. We chose conditions of moderate defocus of images. The CNR was 10 dB, and the target with a radial velocity of 2 m/s was tested. Along with the increase of SCR, the detection probability of the two methods increased. We can also see that GSV-GLRT had a higher detection probability in the low-SCR environment since this method uses adjacent pixels to improve the energy of the target indirectly. The detection performance of the proposed method is closer to its optimal theoretical performance, and better than the GLRT. Thus, the GSV-GLRT reduces the performance loss caused by defocusing and increases the robustness of the system.

#### 4.1.3. Detection Probability Versus Minimum Detectable Velocity

**Figure 5:**
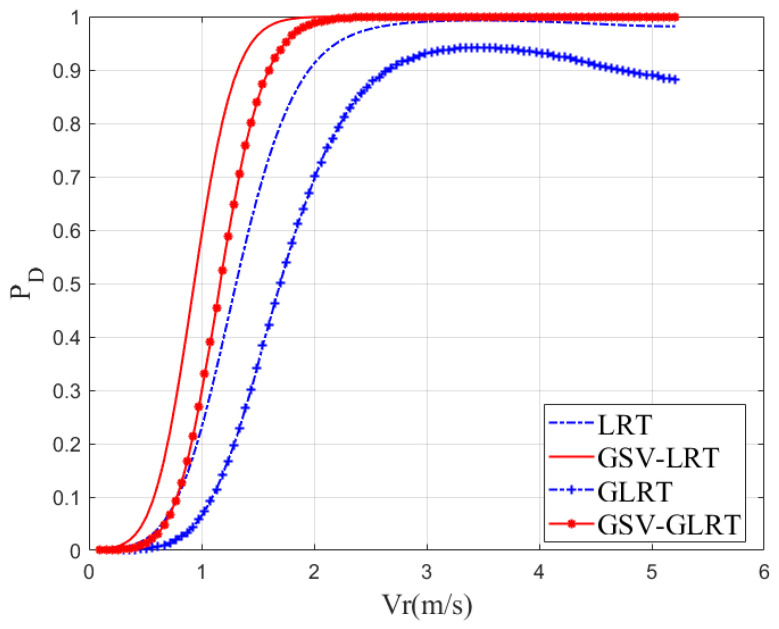
Detection probability versus velocity, SCR = −5 dB, CNR = 13 dB, $P_{FA}=10^{-4}$, three channels, b1 = 0 m, b2 = 0.15 m, b3 = 0.3 m, $\beta ={\left[0.6,0.6,1,0.2,0.2\right]}^{T}⊗1_{3}$.

Next, Figure 5 shows the detection probability of different radial velocities under a constant false alarm probability. The $P_{FA}$ was chosenas under $10^{-4}$. The curves were drafted by using $10^{5}$ repetitions. The CNR was 13 dB and the SCR was −5 dB. We choose the larger velocity range that can be estimated and detected unambiguously to test. When the detection probability was 0.9, theoretically, GSV-LRT can detect a slow-moving target with a velocity of 1.2 m/s, while that of the traditional LRT was 2 m/s. Although the suboptimal GSV-GLRT detection performance declined, it can still be 1.5 m/s, while the traditional GLRT was 2.6 m/s. Therefore, GSV-GLRT is closer to its theoretical optimal performance and has a lower detectable velocity. The reason why GSV-GLRT can reduce the minimum detectable velocity of the system is that it effectively accumulates the moving-target signal. Thus, the GSV-GLRT has the lower minimum detectable velocity of the two.

#### 4.1.4. Cramer Rao Lower Bounds (CRLBs) of the Radial Velocity

**Figure 6:**
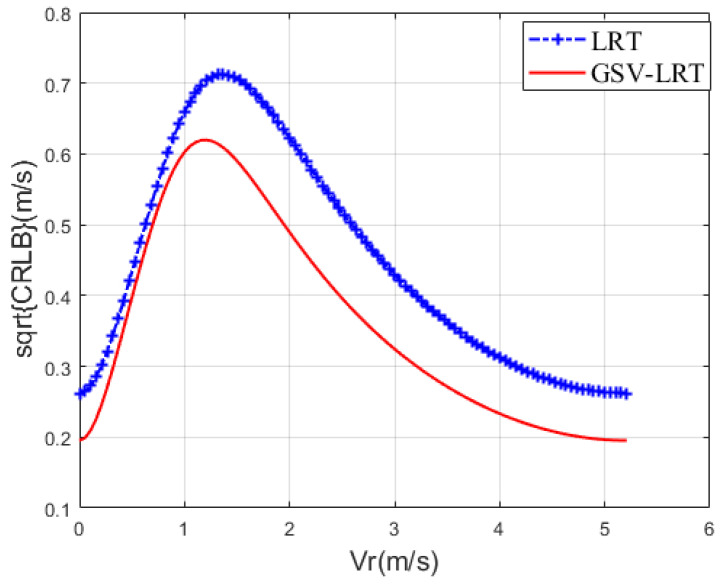
CRBL Curves of the radial velocity, SCR = −5 dB, CNR = 10 dB, three channels, b1 = 0 m, b2 = 0.15 m, b3 = 0.3 m, $\beta ={\left[0.6,0.6,1,0.2,0.2\right]}^{T}⊗1_{3}$.

Lastly, in order to better investigate the estimation accuracy, we report the $CRBL^{1/2}$ curves for the radial velocity in Figure 6 [38]. The CNR is 10 dB and the SCR is -5 dB. Because GSV-GLRT and GLRT do not have a closed expression, CRB cannot be calculated. Comparing GSV-LRT curve with LRT curve, it can be found that the GSV-LRT has better accuracy of radial velocity estimation. In fact, GSV-LRT effectively improves the SCR, which leads to a decrease in CRBL [39]. Accordingly, we can regard that the GSV-GLRT has better accuracy than the GLRT.

### 4.2. Simulated SAR Data

**Figure 7:**
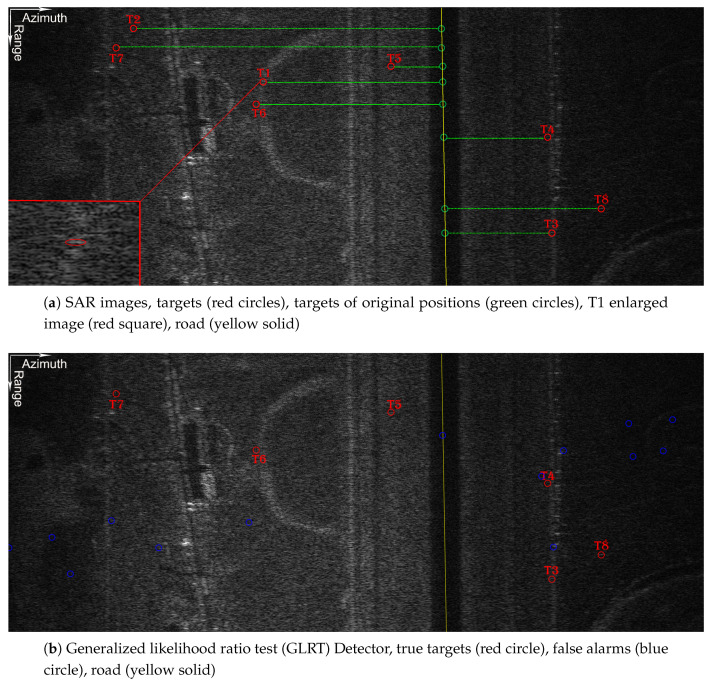

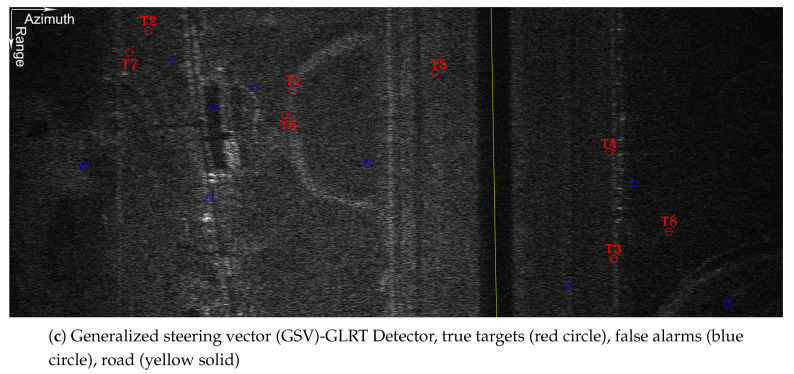
Scene and detection results.

To validate the effectiveness of the GSV-GLRT detector, we conducted experiments by semi-physical simulation. The system and geometry parameters are shown in Table 1. Here, we utilized an airborne SAR image scene (as shown in Figure 7a, the azimuth was 2000 cells and the range was 800 cells) to simulate the echoes of an airborne array radar with 3 channels via point-target simulation (details of the simulation method can be referenced in [40]). Eight injected moving targets were placed in range cells R = 220, 60, 650, 380, 170, 280, 120 and 580. Their parameters are shown in Table 2. Noticing the heterogeneous clutter, we considered local clutter of SCR. In order to simulate the defocus of moving targets, their velocity was set to 0.2 m/s and images were obtained by range-Doppler imaging of static scenes. The scene of the first channel is shown in Figure 7a, and the moving targets are indicated by red circles. Due to the radial velocity, the position of the moving target is displaced and shifted by $\Delta X_{a}$:

(29) $$\Delta X_{a}=-\frac{V_{r}}{V}R$$

where *R* is the slant distance. Once the target velocity is estimated, they can be placed in their original position (green circles).

In Figure 7a, we can observe that T1 is defocusing though it is submerged by the clutter. The defocus of T8 with high SCR is easier to observe. When the false alarm probability was $10^{-4}$, the detection results of the GSV-GLRT are shown in Figure 7c and the detection results of the GLRT are shown in Figure 7b (the points detected near the actual moving target are not regarded as a false alarm point). The detection results show that all the eight moving targets were detected by the GSV-GLRT, while six targets were detected by the GLRT except T1 and T2. The proposed detector improves the detection probability as it reduces the false alarm points. The results of estimated parameters are shown in Table 2. It can be seen from Table 2 that SCR and $V_{r}$ greatly affect the performance of the two detectors, and the detection performance of the moving target with faster radial velocity is better. Slower radial velocities cause smaller phase offsets among channels, and the decreased SCRs caused by defocusing make the detection more difficult. According to the parameter estimation in Table 2, the errors of the GSV-GLRT detector were less than 0.23 m/s, while the errors of the GLRT detector were 1.05 m/s. It can even estimate the parameter of a very slow-moving target within an error of 0.09 m/s.

**Figure 8:**
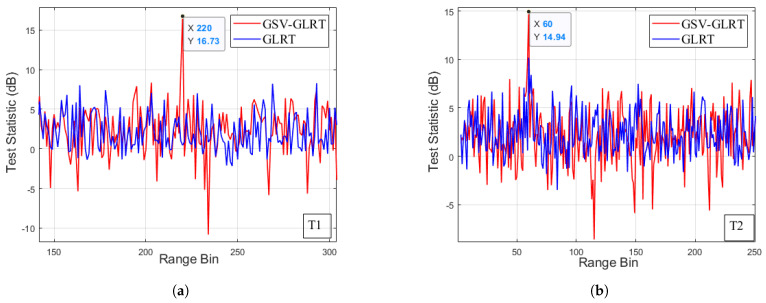
Detection results of the detectors (**a**) test statistic of target T1 in R = 220 (**b**) test statistic of target T2 in R = 60.

Figure 8 shows the test statistics of T1 and T2. The curves of the GSV-GLRT are shown as the red line and the curves of the traditional GLRT are shown as the blue line. For the slow target T1 with SCR = 0 dB and Vr = 0.5 m/s, the estimated error of the GSV-GLRT detector was 0.19 m/s, while the GLRT was invalid. In Figure 8a, for the test statistics of the GSV-GLRT, the target signal was 14 dB above the 3 dB mean value. For the slow target T2 with SCR = 0 dB and Vr = 1 m/s, the estimated error of the GSV-GLRT detector was 0.09 m/s, while the GLRT was invalid. In Figure 8b, for the test statistics of the GSV-GLRT, the target signal was 12 dB above the 3 dB mean value. For the test statistics of the traditional GLRT, the target signal was 7 dB above the 3 dB mean value. However, the highest background peak was close to the target peak. When the SCR increased (i.e., T6 and T7), the performance of the two detectors improved. For the moving targets with low RCS and slow radial velocity, the GSV-GLRT offers a highly improved detection capability, while the performance of the GLRT is severely limited by the velocity and SCR. Moreover, the image defocus may significantly degrade the GLRT performance. In other words, the traditional GLRT is sensitive to the image quality , while the GSV-GLRT is more robust. Obviously, the proposed GSV-GLRT improves the accuracy of parameter estimation. Taken together, the proposed GSV-GLRT outperforms the traditional GLRT in both the detection performance and parameter estimation.

## 5. Discussion

In this study, we proposed an extended GLRT detector of moving targets for multichannel SAR. The results suggest a possibility of improving detection performance by combining adjacent pixels. We derive the GSV-GLRT detector based on the joint data vector and the joint covariance matrix. In the case of the known target parameters, we also derive the GSV-LRT in a closed form as an optimal reference. In practical applications, the GSV-GLRT is utilized because the target parameters are unknown. Compared with the optimal GSV-LRT, its performance degrades due to the parameter estimation inaccuracies. Compared with the traditional GLRT, the GSV-GLRT offers a dramatic improvement in detection performance and reaches an accurate estimation of the target radial velocity. The errors of the GSV-GLRT detector were less than 0.23 m/s, while the errors of the GLRT detector were 1.05 m/s. In particular, for the slow-moving target with radial velocity of 0.5 m/s, the signal intensity improved by approximately 15 dB and the error of the estimated parameter was 0.19 m/s over the traditional GLRT.

The main purpose of the detector is to achieve moving-target detection in defocused SAR images. Although this study demonstrates important progress, some limitations are also noteworthy. First, the covariance matrix is unknown in practical applications and must be estimated by using adaptive techniques [9,29,34,41]. The GSV-GLRT is based on the generalized steering vector, which increases the joint processing dimension. Therefore, the training data size increases dramatically, which can lead to excessive training and computational burden. Second, the performance of the GSV-GLRT benefits from the selection of approrpiate windows of the generalized steering vector. Further improvement may be possible by adaptively selecting proper windows for processing, which is a topic for future studies. Moreover, it is meaningful to analyze and evaluate the performance of the GSV-GLRT in different realistic scenarios.

## 6. Conclusions

In the complex image domain, inevitable image defocus typically produces a loss in the signal intensity, which severely degrades the performance of the moving-target detectors. To address this issue, this paper proposes an extended GLRT detector of moving targets for multichannel SAR. A Gaussian clutter model and a deterministic target response are assumed. Then, based on the generalized steering vector, the modified signal statistical model was established, and the theory of the GSV-GLRT and its mathematical framework were introduced. The detector mainly accumulated the dispersed energy of the targets while the distribute was suppressed, and then achieves the target detection. Both theoretical analysis and SAR data of the semi-physical simulation were carried out to verify the effectiveness of the GSV-GLRT detector. The results show that, compared with the previous GLRT, the proposed GSV-GLRT significantly improves the detection performance and accurately estimate the target radial velocity. It will be very useful to detect the moving targets in defocused SAR images.

## Figures and Tables

**Table 1:** System and geometry parameters.

Parameters	Variables	Values
Scene dimensions	Na × Nr	2000 × 800
Wavelength	λ	0.03 m
Platform velocity	V	100 m/s
Number of channels	N	3
Antenna separation	b	0.3 m
Pulse repetition frequency	PRF	1000 Hz
Platform Height	Ha	3000 m
Minimum slant range	Rmin	6000 m
Bandwidth	Br	30 MHz
Clutter to noise ratio	CNR	20 dB
False alarm probability	$P_{FA}$	0.01%

**Table 2:** Target Parameters and Estimated Parameters.

ID	SCR (dB)	Radial Velocity (m/s)	GLRT Estimated Velocity (m/s)	GLRT Estimated Error (m/s)	GSV-GLRT Estimated Velocity (m/s)	GSV-GLRT Estimated Error (m/s)
T1	0	0.5	–	–	0.31	0.19
T2	0	1	–	–	1.09	0.09
T3	−2	2.5	3.55	1.05	2.27	0.23
T4	−2	3	3.11	0.11	3.03	0.03
T5	−2	4	4.03	0.03	4.21	0.21
T6	5	0.5	0.52	0.02	0.47	0.03
T7	5	1	1.57	0.57	0.96	0.04
T8	25	2.5	2.53	0.03	2.46	0.04

Note: - denotes unidentified target.

## Data Availability

Not applicable.

## Abbreviations

The following abbreviations are used in this manuscript:

GLRT	Generalized likelihood ratio test
LRT	Likelihood ratio test
GSV	Generalized steering vector
RCS	Radar cross section
CFAR	Constant false alarm rate
DPCA	Displaced phase center antenna
ATI	Along-track interferometry
SCR	Signal to clutter ratio
CNR	Clutter to noise ratio
PDF	Probability density function
